# Left Atrial Appendage Occlusion in Patients with Failure of Antithrombotic Therapy: Good Vibes from Early Studies

**DOI:** 10.3390/jcm12113859

**Published:** 2023-06-05

**Authors:** Alberto Preda, Matteo Baroni, Marisa Varrenti, Sara Vargiu, Marco Carbonaro, Federica Giordano, Lorenzo Gigli, Patrizio Mazzone

**Affiliations:** Cardio-Thoraco-Vascular Department, Electrophysiology Unit, ASST Grande Ospedale Metropolitano Niguarda, 20162 Milan, Italy

Atrial fibrillation (AF) is the most common sustained cardiac arrhythmia and predisposes patients to an increased risk of cardioembolic events (CE), such as ischemic stroke, TIA, or systemic embolism [1]. Oral anticoagulants (OACs), including direct oral anticoagulants (DOACs) and vitamin K antagonists (VKA), proved to effectively prevent the majority of these events [2]. In patients who experienced an event while on OACs, the recurrence rate of CE is twice as high compared to those who were not anticoagulated at the time of the index event [3]. In non-valvular AF patients, up to 98% of thrombi are located in the left atrial appendage (LAA) [4], and this finding is rather common (17%) during transesophageal echocardiography, cardiac surgery, and autopsy [5]. The presence of LAA sludge or thrombosis in patients with adequate OAC therapy must be considered as a case of OAC failure, equally related to a cardioembolic event, since its predictive role in clinical CE has been demonstrated [6]. In a large observational left atrial appendage occlusion (LAAO) registry, including patients with high-risk AF, the annual CE rate reported was 2% [7]. Therefore, in this group at particularly high risk of CE recurrence, further prevention strategies are deemed necessary. 

Clinical guidelines do not provide clear recommendations for the management of patients with evidence of OAC failure. Indeed, the current European guidelines do not recommend switching from one DOAC to another, nor to intensify anticoagulation strategies [8]. Several studies reported that intensification of OAC in order to achieve thrombus resolution/effective secondary prevention is associated with a suboptimal result and a concomitant increased bleeding risk [9,10]. Percutaneous catheter-based devices have been developed to exclude the LAA from systemic circulation. LAA occlusion (LAAO) is currently indicated in the case of high bleeding risk or contraindication to OAC therapy [8]. However, this invasive strategy may also play a key role in patients where OAC therapy failed, as reported in the recent expert consensus statement of EHRA/EAPCI [11]. Whether the addition of LAAO to prolonged OAC increases the efficacy of CE prevention in patients with prior embolic event or presenting with LAA sludge, despite appropriate OAC therapy, is currently a matter of debate. Although published studies are still few, with not-negligible limitations and underpowered factors to determine the efficacy of this strategy, the results in terms of safety seem to be promising. An analysis of the Amplatzer Cardiac Plug multicenter registry, which compared patients with previous stroke on OAC treated with LAAO and patients with other indications for occlusion, showed similar safety outcomes, as well as a significant reduction in stroke/TIA recurrence and major bleeding events during a mean follow-up (FU) of 16 months [12]. Only one group-controlled study retrospectively compared patients who experienced a CE, despite adequate DOAC therapy. LAAO was also performed versus patients who only continued DOACs. After a median FU of 3.4 years, the LAAO group showed a 72% reduction in a composite of CEs, major bleeding, or procedure-related major complications [13]. 

LAAO is relatively contraindicated in the presence of LAA thrombosis, since these patients were excluded in large studies [14]. Indeed, performing LAAO in the presence of LAA sludge or thrombosis is associated with a potential risk of peri-procedural CEs, namely, stroke, although that was not proved. On the contrary, the most recent systematic review on LAAO in the presence of LAA thrombus, which included 16 studies with 58 patients recruited, reported only one stroke and two device-related thromboses during a mean FU of 3.4 months [15]. In this cohort, 65% of patients presented a contraindication to OAC therapy, while 35% showed OAC therapy failure. The median CHADsVASc and HAS-BLED scores were 4 and 3, respectively. All cases underwent successful implantation of LAAO devices. The Amplatzer Amulet (Abbott) was the most commonly used device (50%). In the largest multicenter observational study published so far [10], including 126 patients with LAA thrombus on procedural imaging referred for LAAO, Marroquin et al. demonstrated that patients who underwent LAAO showed lower stroke rate compared to a control group with intensified anticoagulant therapy (no CE vs. 2.9%) at a mean follow-up of 18 months. Procedural success was 90.5%. In the OAC intensification group, total thrombus resolution was observed early in 60.3% of patients and, later, in 75.3% of patients. 

The feasibility and the safety of LAAO in the presence of thrombus was assessed in the multicenter retrospective registry TRAPEUR, where 53 patients (3%) underwent LAAO with Amplatzer Amulet (86%) and Watchman FLX (14%) in the presence of LAA thrombosis [16]. Only one patient met the primary endpoint of 30-day occurrence of stroke, CE, or cardiovascular death. A transesophageal echocardiography image of LAAO with Watchman FLX in the presence of LAA thrombosis is represented in Figure 1.

Moreover, the long-term safety of the LAAO in a mixed population with previous CE or LAA thrombosis was reported in a smaller study with a mean follow-up of 47.2 months [17].

To further reduce the risk of CE during the procedure, the use of cerebral embolic protection devices is rapidly gaining traction [18]. These are filters designed to capture or deflect emboli traveling to the brain during the procedure. They are normally positioned across the origin of supra-aortic vessels at the beginning of the procedure and are retrieved at its end. Figure 2 shows the fluoroscopic image of the most frequently used cerebral embolic protection device (TriGUARD 3 CEP, Keystone Heart). While their role in transcatheter aortic valve replacement (TAVR) is more defined [19], clear data on their safety and efficacy in the context of LAAO are still lacking because less than 30% of the recruited patients in the abovementioned studies employed such devices. The choice regarding post-LAAO antithrombotic therapy is also of paramount importance. Clinical guidelines do not establish clear recommendations on how to manage patients who suffer recurrent CEs on OACs, besides assessing good therapeutic adherence. Indeed, it is not advised to either switch from one OAC therapy to another, nor to intensify anticoagulant therapies (e.g., supratherapeutic INRs, dual, or triple antithrombotic therapy). In clinical practice, several strategies are reported, such as unchanging OAC therapy, adding an antiplatelet drug, changing the DOAC, or switching DOAC to LMWH or VKA if the patient was on DOAC before the index event, with variable efficacy among studies [3,8]. In patients at very high thrombotic risk, LAAO may be essentially considered as an enhancement of OAC therapy. However, no consensus suggests whether to continue OAC therapy after LAAO, nor how to modify it. Even if large variability exists among retrospective studies, preliminary data show beneficial effects of OAC therapy continuation vs OAC interruption. In the metanalysis of Sharma et al., the majority of cases continued OAC therapy or switched to dual antiplatelet therapy after LAAO [15]. Bleeding complications occurred more frequently in patients who intensified anticoagulant therapy compared to those who performed LAAO and continued OAC therapy (10.5% vs. 22.5%, respectively [*p* = 0.102]). In a study by Marroquin et al., bleeding complications during intensified anticoagulant therapy occurred in 9.6% of patients, compared to 3.8% of patients who performed LAAO and continued the same OAC [10]. Margonato et al. demonstrated the independent protective role of maintaining lifelong OAC therapy vs. terminating it after LAAO, as well as a positive trend towards being survival-free regarding all-cause death, systemic stroke, CE, and major bleeding provided by this strategy [17]. Patients who may be candidates for LAAO historically include those with a previous history of bleeding complications on OAC, those with an increased risk of bleeding, and those who are unable to comply with OAC therapy. More recently, the possible use of LAAO in the synergy of OAC therapy in patients with extremely high embolic risk, such as those with evidence of failure of OAC therapy, has been proposed [20]. Despite the need for prospective randomized studies on this topic, current data have provided positive results, especially in terms of procedural safety. Moreover, further studies are needed to establish the appropriate antithrombotic strategy after LAAC. 

## Figures and Tables

**Figure 1:**
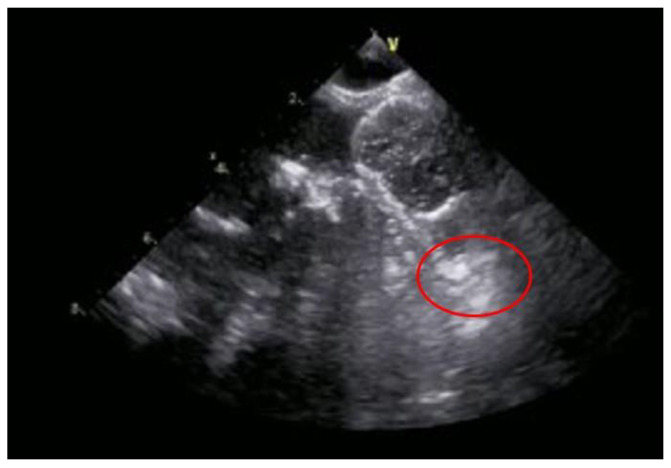
Transesophageal echocardiography image of LAAO with Watchman FLX in the presence of LAA thrombosis.

**Figure 2:**
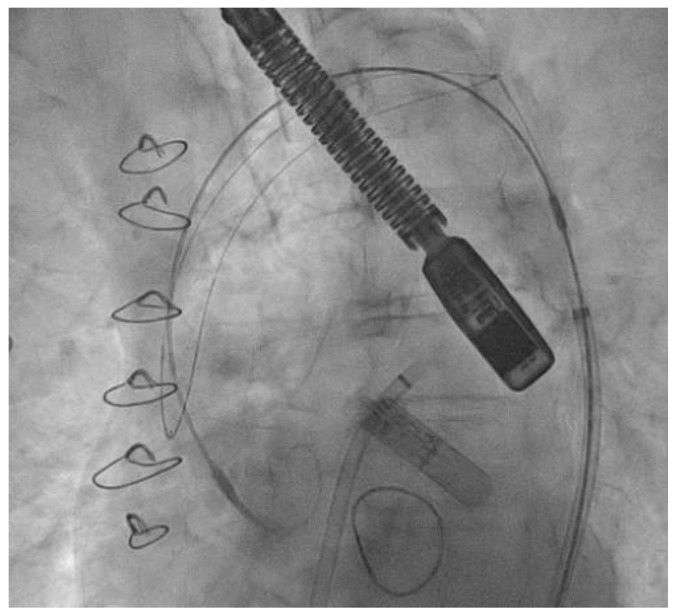
Fluoroscopic image of TriGUARD 3 CEP once released across the origin of the supra-aortic vessels.

## References

[1] Lip G.Y. (1995). Does atrial fibrillation confer a hypercoagulable state?. Lancet.

[2] Hart R.G., Pearce L.A., Aguilar M.I. (2007). Meta-analysis: Antithrombotic therapy to prevent stroke in patients who have nonvalvular atrial fibrillation. Ann. Intern. Med..

[3] Seiffge D.J., De Marchis G.M., Koga M., Paciaroni M., Wilson D., Cappellari M., Macha K., Tsivgoulis G., Ambler G., Arihiro S. (2020). Ischemic Stroke despite Oral Anticoagulant Therapy in Patients with Atrial Fibrillation. Ann. Neurol..

[4] Manning W.J., Weintraub R.M., Waksmonski C.A., Haering J.M., Rooney P.S., Maslow A.D., Johnson R.G., Douglas P.S. (1995). Accuracy of transesophageal echocardiography for identifying left atrial thrombi. A prospective, intraoperative study. Ann. Intern. Med..

[5] Blackshear J.L., Odell J.A. (1996). Appendage obliteration to reduce stroke in cardiac surgical patients with atrial fibrillation. Ann. Thorac. Surg..

[6] Lowe B.S., Kusunose K., Motoki H., Varr B., Shrestha K., Whitman C., Tang W.W., Thomas J.D., Klein A.L. (2014). Prognostic significance of left atrial appendage “sludge” in patients with atrial fibrillation: A new transesophageal echocardiographic thromboembolic risk factor. J. Am. Soc. Echocardiogr..

[7] Boersma L.V., Ince H., Kische S., Pokushalov E., Schmitz T., Schmidt B., Gori T., Meincke F., Protopopov A.V., Betts T. (2019). Evaluating Real-World Clinical Outcomes in Atrial Fibrillation Patients Receiving the WATCHMAN Left Atrial Appendage Closure Technology: Final 2-Year Outcome Data of the EWOLUTION Trial Focusing on History of Stroke and Hemorrhage. Circ. Arrhythmia Electrophysiol..

[8] Hindricks G., Potpara T., Dagres N., Arbelo E., Bax J.J., Blomström-Lundqvist C., Watkins C.L. (2020). 2020 ESC Guidelines for the diagnosis and management of atrial fibrillation developed in collaboration with the European Association for Cardio-Thoracic Surgery (EACTS): The Task Force for the diagnosis and management of atrial fibrillation of the European Society of Cardiology (ESC) Developed with the special contribution of the European Heart Rhythm Association (EHRA) of the ESC. Eur. Heart J..

[9] Tarantini G., D’Amico G., Latib A., Montorfano M., Mazzone P., Fassini G., Maltagliati A., Ronco F., Sacca S., Cruz-Gonzalez I. (2018). Percutaneous left atrial appendage occlusion in patients with atrial fibrillation and left appendage thrombus: Feasibility, safety and clinical efficacy. EuroIntervention.

[10] Marroquin L., Tirado-Conte G., Pracoń R., Streb W., Gutierrez H., Boccuzzi G., Arzamendi-Aizpurua D., Cruz-González I., Ruiz-Nodar J.M., Kim J.S. (2022). Management and outcomes of patients with left atrial appendage thrombus prior to percutaneous closure. Heart.

[11] Michael G., Wolff R., Hindricks G., Mandrola J., Camm A.J., Lip G.Y., Fauchier L., Betts T.R., Lewalter T., Saw J. (2020). EHRA/EAPCI expert consensus statement on catheter-based left atrial appendage occlusion—An update. EuroIntervention.

[12] Cruz-González I., González-Ferreiro R., Freixa X., Gafoor S., Shakir S., Omran H., Berti S., Santoro G., Kefer J., Landmesser U. (2020). Left atrial appendage occlusion for stroke despite oral anticoagulation (resistant stroke). Results from the Amplatzer Cardiac Plug registry. Rev. Española Cardiol..

[13] Falasconi G., Gaspardone C., Godino C., Gaspardone A., Radinovic A., Pannone L., Leo G., Posteraro G.A., Slavich M., Melillo F. (2021). Left atrial appendage closure: A new strategy for cardioembolic events despite oral anticoagulation. Panminerva Med..

[14] Piccini J.P., Sievert H., Patel M.R. (2017). Left atrial appendage occlusion: Rationale, evidence, devices, and patient selection. Eur. Heart J..

[15] Sharma S.P., Cheng J., Turagam M.K., Gopinathannair R., Horton R., Lam Y.Y., Tarantini G., D’Amico G., Freixa Rofastes X., Lange M. (2020). Feasibility of Left Atrial Appendage Occlusion in Left Atrial Appendage Thrombus: A Systematic Review. JACC Clin. Electrophysiol..

[16] Sebag F.A., Garot P., Galea R., De Backer O., Lepillier A., De Meesteer A., Hildick-Smith D., Armero S., Moubarak G., Ducrocq G. (2022). Left atrial appendage closure for thrombus trapping: The international, multicentre TRAPEUR registry. EuroIntervention.

[17] Margonato D., Preda A., Ingallina G., Rizza V., Fierro N., Ancona F., Agricola E., Bella P.D., Mazzone P. (2023). Left atrial appendage occlusion after thromboembolic events or left atrial appendage sludge during anticoagulation therapy: Is two better than one? Real-world experience from a tertiary care hospital. J. Arrhythmia.

[18] Berg J., Preda A., Fierro N., Marzi A., Radinovic A., Bella P.D., Mazzone P. (2023). A Referral Center Experience with Cerebral Protection Devices: Challenging Cardiac Thrombus in the EP Lab. J. Clin. Med..

[19] Stachon P., Kaier K., Heidt T., Wolf D., Duerschmied D., Staudacher D., Zehender M., Bode C., von Zur Mühlen C. (2021). The Use and Outcomes of Cerebral Protection Devices for Patients Undergoing Transfemoral Transcatheter Aortic Valve Replacement in Clinical Practice. JACC Cardiovasc. Interv..

[20] Kaczmarek K., Cygankiewicz I., Streb W., Plaksej R., Jakubowski P., Kalarus Z., Ptaszynski P., Wranicz J.K., Babicz-Sadowska A., Markiewicz A. (2021). Percutaneous Occlusion of the Left Atrial Appendage with Thrombus Irresponsive to Antithrombotic Therapy. J. Clin. Med..

